# Median Nerve Variation: A Complete Spin before Terminal Branching

**DOI:** 10.1055/s-0039-3402734

**Published:** 2020-02-12

**Authors:** Amgad S. Hanna, Zhikui Wei, Barbara A. Hanna

**Affiliations:** 1 Department of Neurological Surgery, University of Wisconsin School of Medicine and Public Health, Madison, Wisconsin, United States; 2 Department of Neurology, Vanderbilt University, Nashville, Tennessee, United States; 3 College of Letter and Science (Premed), Undergraduate Student, University of Wisconsin, Madison, Wisconsin, United States

**Keywords:** anatomy, carpal tunnel, flexor digitorum superficialis, median nerve, variants

## Abstract

Median nerve anatomy is of great interest to clinicians and scientists given the importance of this nerve and its association with diseases. A rare anatomical variant of the median nerve in the distal forearm and wrist was discovered during a cadaveric dissection. The median nerve was deep to the flexor digitorum superficialis (FDS) in the carpal tunnel. It underwent a 360-degree spin before emerging at the lateral edge of FDS. The recurrent motor branch moved from medial to lateral on the deep surface of the median nerve, as it approached the distal carpal tunnel. This variant doesn't fall into any of Lanz's four groups of median nerve anomalies. We propose a fifth group that involves variations in the course of the median nerve. This report underscores the importance of recognizing variants of the median nerve anatomy in the forearm and wrist during surgical interventions, such as for carpal tunnel syndrome.

## Introduction

Median nerve anatomy and variations have fascinated both clinicians and researchers. Classically, the median nerve is described to be derived from both the lateral and medial cords of the brachial plexus. It runs down the upper arm without any branches, and enters forearm between the two heads of pronator teres. In the forearm, the median nerve travels between flexor digitorum superficialis (FDS) and flexor digitorum profundus. It then gives out the anterior interosseous nerve from the dorsolateral aspect. In the wrist and hand, median nerve travels deep to the flexor retinaculum (transverse carpal ligament) and gives out its terminal branches, including the recurrent motor branch on the lateral side, and the palmar digital branches medially.

While textbook descriptions provide an excellent baseline for understanding the anatomy of this nerve, it does not represent the whole reality of median nerve anatomy, as seen by surgeons and researchers. Median nerve anatomical variations have been frequently reported in the literature.

We report a rare median nerve anatomical variant that was encountered during a cadaveric dissection. The median nerve was deep to FDS in the carpal tunnel and showed a 360-degree spin before dividing into its terminal branches. To our knowledge, this has never been described before. Recognition is a key since this anomaly may put the median nerve at risk of injury during surgery for carpal tunnel release.

## Case Presentation

**Fig. 1:**
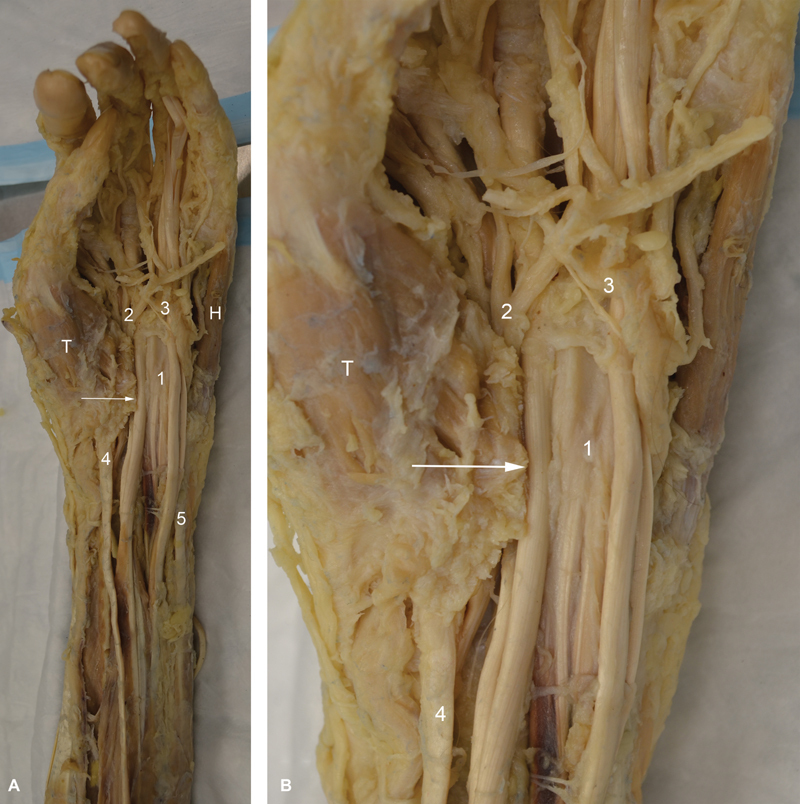
( **A** ) Dissection of the palmar aspect of the left hand and forearm. Only tendons of flexor digitorum superficialis (FDS; 1) were visible once the flexor retinaculum (transverse carpal ligament) was opened. The median nerve, while not visible in the carpal tunnel, its branches (2) appeared distally toward the radial aspect of FDS and crossed medially superficial to FDS along with the superficial palmar arch (3). (4) represents flexor carpi radialis and (5) is the flexor carpi ulnaris. H, hypothenar muscles; T, thenar muscles; arrow, cut end of the transverse carpal ligament. ( **B** ) Magnified view of A.

**Fig. 2:**
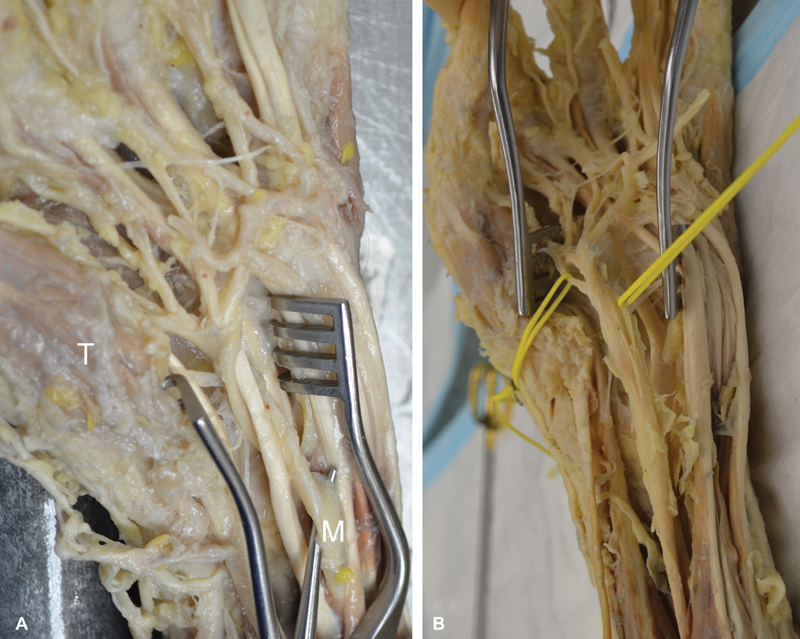
( **A** ) When the flexor tendons were retracted, the median nerve (M) was observed making a 360-degree spiral turn deep to the FDS tendons to emerge lateral to then superficial to FDS. ( **B** ) Interfascicular dissection showing the palmar recurrent branch (yellow vessel loops) moving from medial to lateral on the deep surface of the median nerve toward its destination to the thenar muscles. FDS, flexor digitorum superficialis; T, thenar muscles.

**Fig. 3:**
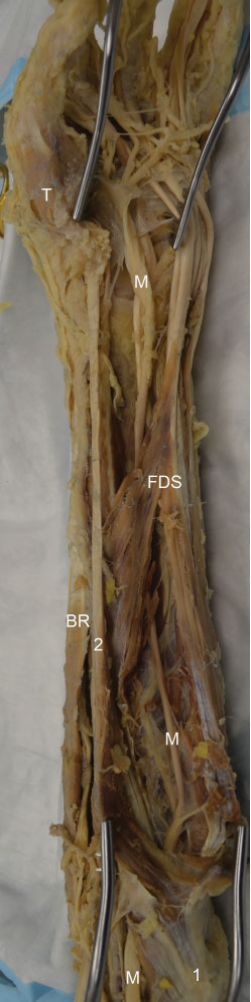
The entire course of the median nerve is observed from the cubital fossa, under the flexor muscles, and then distally making a 360-degree turn until it branches distal to the transverse carpal ligament. 1, common flexor tendon originating from the medial epicondyle of the humerus; 2, flexor carpi radialis; BR, brachioradialis; FDS, flexor digitorum superficialis; M, median nerve; T, thenar muscles.

This is a cadaveric dissection of an 82-year-old female's left upper limb. There were no signs of trauma or previous surgery. Once the flexor retinaculum was opened, only tendons of FDS were seen ( Fig. 1 ). Normally the median nerve is located between the flexor retinaculum and the FDS. Further dissection revealed that the median nerve made a 180-degree spin around the lateral border of the FDS tendon to the index finger toward the distal end of the carpal tunnel ( Figs. 1 and 2 ). The median nerve and its branches then travelled medially superficial to FDS, along with the superficial palmar arch ( Figs. 1 and 2 ). When the FDS tendons were separated, the median nerve was found to have another 180-degree spin around itself proximal to the carpal tunnel deep to FDS, thus completing a 360-degree spin ( Fig. 2A ). An interfascicular dissection was then performed to separate the palmar recurrent branch. This was found to be on the medial aspect of the median nerve in the proximal carpal tunnel, and then it travels on the deep surface of the median nerve to head laterally toward the thenar muscles in the distal carpal tunnel ( Fig. 2B ). We completed the dissection by exposing the median nerve in the cubital fossa and the upper forearm ( Fig. 3 ).

## Discussion

Carpal tunnel syndrome is one of the most common forms of entrapment neuropathy, often requiring surgical release of the transverse carpal ligament. Common causes of carpal tunnel syndrome include changes that led to the increased tissue turgor, hypertrophy of connective tissue, or deposition of metabolic products in the tissue. These changes could be the downstream effects of traumatic, hormonal, metabolic, vascular, inflammatory, neoplastic, or aging processes.

Stancić et al studied 100 hands and found that less than 50% of them aligned with the median nerve anatomy described in textbooks. 1 Thus, they proposed that knowledge of these variations should be applied preoperatively to minimize chances of incomplete decompression of the nerve during surgery. Henry et al proposed that variations in median nerve location and branching in the carpal tunnel are not only existing but are common, and carpal tunnel release should be approached from the ulnar side to minimize further injury in surgery. 2 Vinding et al identified a rare anatomical variant of the thenar branch that originates from ulnar side and runs supraligmentously close to the top of the transverse ligament which carries great risk of injury during endoscopic release for carpal tunnel syndrome. 3 Hanna classified the motor branch of the median nerve into types I-IV based on location on the median nerve, and A-D based on the angle it takes. 4 Spagnoli et al reported a high division of the median nerve proximal to the carpal tunnel, also known as a bifid median nerve, and its association with carpal tunnel syndrome and with persistent median vessels. 5 Lis et al reported a variation of the median nerve that passed through the head of the flexor digitorum superficialis. 6 Atoni and Oyinbo identified a splitting of the median nerve into medial and lateral divisions in the proximal one-third of the forearm to accommodate an anomalous muscle which could be a source of median nerve compression. 7 Papathanassiou found a variation of the median nerve that gave a motor branch more proximally than expected, thus passing through the flexor retinaculum. 8

Lanz supported the ulnar approach in surgery, reporting 29 variations in 246 hands. He classified the variations of median nerve in the wrist into four groups. Group I includes variations of the thenar branch. Group II includes variations that involve accessory branches of the median nerve at the distal end of carpal tunnel. Group III includes median nerve with high divisions, and group IV includes variations that involve accessory branches proximal to the carpal tunnel. 9 Among group I, four variations are identified and these include subligamentous, transligamentous, ulnarwards, and supraligamentous courses of thenar branch, in addition to the standard anatomy, extraligamenous thenar branch. 9 Interestingly, our case identified an anatomical basis for the ulnarward origin of the thenar branch which is due to a proximal rotation of median nerve deep to FDS rather than actual variations of the terminal branches of median nerve itself. This warrants adding a separate group, may be group V that includes variation in the course of the median nerve.

This case highlights the importance of recognizing variations of the median nerve anatomy in the forearm and wrist region, and their significance with regard to the diagnosis and treatment of conditions, such as the carpal tunnel syndrome. It is crucial to gather as much information as possible regarding the anatomy prior to surgery, as well as be able to recognize the variations when encountered intraoperatively, to minimize the possibility of iatrogenic events.
